# Admission HDL-C and recurrence risk of hypertriglyceridemia-induced acute pancreatitis: a multicenter cohort study

**DOI:** 10.3389/fnut.2026.1741265

**Published:** 2026-01-30

**Authors:** Jianying Liu, Haimei Xu, Chenchen Huang, Xiaonan Qiu, Xuefeng Lu, Chengzhao Weng, Chao Wang, Yijiao Xu

**Affiliations:** 1 Zhongshan Hospital (Xiamen), Fudan University, Xiamen, China; 2 Zhangzhou Municipal Hospital of Fujian Province, Zhangzhou, China; 3 900th Hospital of PLA Joint Logistic Support Force, Fuzhou, China

**Keywords:** acute pancreatitis, high-density lipoprotein cholesterol, hypertriglyceridemia, recurrent acute pancreatitis, risk factor

## Abstract

**Background:**

Hypertriglyceridemia-induced acute pancreatitis (HTG-AP) carries a high risk of post-discharge recurrence. Whether admission high-density lipoprotein cholesterol (HDL-C) independently predicts recurrence and how its effect varies over time remain unclear.

**Methods:**

A multicenter retrospective study was conducted, including consecutive HTG-AP inpatients from three hospitals from January 2020 to March 2025. The primary endpoint was overall post-discharge recurrence; while the secondary endpoints included recurrence within 6 and 12 months. HDL-C, analyzed both as a continuous variable and by tertiles, was evaluated using Cox models with progressive adjustment. Dose–response was examined by restricted cubic splines (RCS). Time-varying effects were assessed with landmark analyses (0–6 and 6–12 months) and an HDL × log(time) interaction. Robustness was tested using inverse probability of treatment weighting (IPTW). Restricted mean time free of recurrence (RMST) quantified absolute differences.

**Results:**

A total of 440 patients were enrolled, and the median follow-up duration was 14.0 months. Lower admission HDL-C was associated with a higher recurrence risk. In the fully adjusted model, each 1 mmol/L increase in HDL-C was associated with a 77% lower hazard of overall recurrence (HR 0.23, 95% CI 0.11–0.48; *p* < 0.001). Compared with the lowest tertile (T1), risks were reduced in T2 (HR 0.42, 95% CI 0.27–0.64) and T3 (HR 0.33, 95% CI 0.19–0.55; *p* for trend<0.001). RCS showed a clear linear dose–response with risk plateauing at higher HDL-C. Landmark analyses localized the predictive value to the first year: 0–6 months HR 0.045 (95%CI: 0.012–0.171; *p* < 0.001) and 6–12 months HR 0.176 (95%CI: 0.054–0.568; *p* = 0.004); no association was observed beyond 12 months (*p* = 0.197). RMST within 12 months was 2.2 months shorter in T1 and 0.5 months shorter in T2 versus T3. Findings were consistent after IPTW (0–6 months aHR = 1.76, 95%CI: 1.12–2.77; 6–12 months aHR = 2.27, 95%CI: 1.38–3.73; 12-month aHR = 3.79, 95%CI: 1.92–7.48; overall aHR = 1.80, 95%CI: 1.14–2.83).

**Conclusion:**

Low admission HDL-C is an independent predictor of HTG-AP recurrence with a linear risk gradient, and its predictive effect is most pronounced within 12 months after discharge. Given its stability and accessibility, HDL-C can aid early identification of high-risk patients and inform targeted follow-up and prevention strategies.

## 1 Background

Acute pancreatitis (AP) is one of the most frequent acute inflammatory diseases of the digestive tract, with an incidence that has been steadily increasing, imposing a heavy burden on public health and healthcare systems (1). In recent years, due to shifts in dietary patterns, increasing obesity, and a growing population with metabolic diseases, the proportion of acute pancreatitis cases attributed to hypertriglyceridemia (HTG-AP) has increased annually, rising from less than 15% around the year 2000 to over 30% in recent years, making it the second leading cause of AP after gallstones (2, 3). In contrast to biliary or alcohol-related AP, patients with HTG-AP are predominantly young men who frequently exhibit metabolic abnormalities such as obesity and diabetes. They also have a higher tendency for recurrence, which imposes lasting effects on long-term health and quality of life (3–5). Consequently, early detection and intervention for patients at high risk of HTG-AP recurrence represent a critical priority in current clinical practice.

Lipid metabolic disturbances are crucial contributors to both the initiation and progression of HTG-AP (6). Triglycerides are central to its pathogenesis; however, triglyceride levels measured at presentation may decline rapidly with fasting and early management, which can limit the utility of a single admission value as a surrogate for longer-term post-discharge metabolic control (7, 8). Beyond hypertriglyceridemia, abnormalities in the concentration and function of high-density lipoprotein cholesterol (HDL-C) also warrant attention. HDL-C is a multifunctional lipoprotein involved in lipid transport and reverse cholesterol transport and exerts anti-inflammatory, antioxidant, and endothelial homeostatic effects (9, 10). In acute pancreatitis, lower HDL-C levels have been associated with longer hospital stay (11), higher rates of organ failure (12, 13), and increased mortality (14). In this context, admission HDL-C may capture a broader metabolic–inflammatory phenotype and complement triglycerides for risk stratification; however, its association with post-discharge recurrence risk in HTG-AP remains poorly characterized.

Based on this rationale, this study utilized a multicenter retrospective cohort to comprehensively assess the relationship between admission HDL-C levels and the recurrence risk of HTG-AP, with the goal of uncovering the potential role of HDL-C in long-term disease management and offering new clinical insights for early detection and precision intervention in high-risk individuals.

## 2 Methods and materials

### 2.1 Study population

This study was a multicenter retrospective cohort including patients diagnosed with HTG-AP who were hospitalized between January 2020 and March 2025 at Zhongshan Hospital (Xiamen), Fudan University, the 900th Hospital of Joint Logistics Support Force of Chinese People’s Liberation Army (PLA), and Zhangzhou Municipal Hospital of Fujian Province.

Inclusion criteria: eligible patients had confirmed HTG-AP during the index hospitalization and were either first-episode cases or had an adequately documented history of prior pancreatitis episodes.

**Figure 1:**
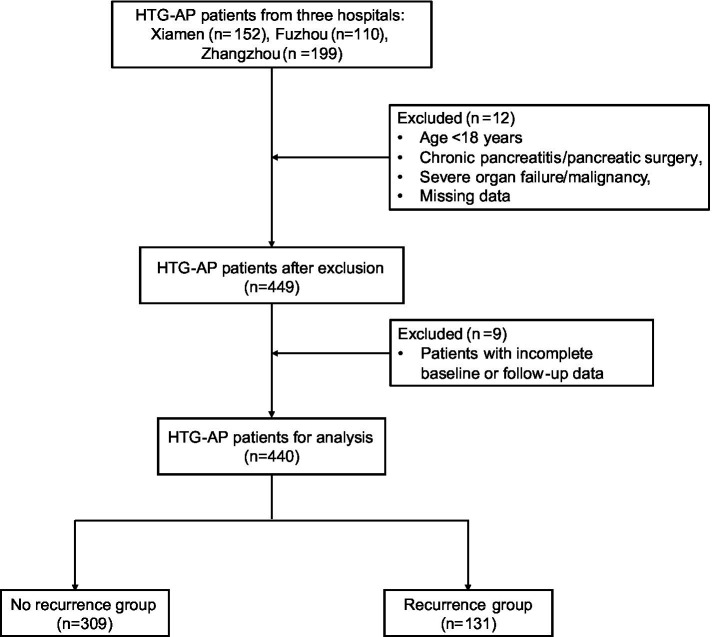
Flowchart of patient selection in the multicenter cohort study.

Exclusion criteria: (1) age <18 years; (2) confirmed chronic pancreatitis; (3) history of pancreatic surgery; (4) severe cardiac, hepatic, or renal insufficiency; (5) malignancy; (6) pregnancy or lactation; and (7) missing essential clinical or follow-up data. The study flowchart is shown in Figure 1.

This study was approved by the Ethics Committees of Zhongshan Hospital (Xiamen) Fudan University (B2025-026), 900th Hospital of PLA Joint Logistic Support Force (2025–068), and Zhangzhou Municipal Hospital (2025KYZ249).

### 2.2 Definitions

Diagnosis of acute pancreatitis was established according to the Guidelines for diagnosis and treatment of acute pancreatitis in China (2021, 15), requiring at least two of the following criteria: (1) characteristic symptoms of acute pancreatitis such as acute upper abdominal pain; (2) serum amylase or lipase levels elevated to more than three times the upper limit of normal; and (3) imaging findings consistent with acute pancreatitis. Diagnosis of HTG-AP was made if serum triglyceride levels were ≥11.3 mmol/L at presentation, or between 5.65 and 11.3 mmol/L after excluding other possible causes. The severity of disease was graded based on the 2012 revised Atlanta classification and definitions: mild acute pancreatitis—no organ failure or local/systemic complications; moderately severe acute pancreatitis—local/systemic complications or transient organ failure (≤48 h); and severe acute pancreatitis—persistent organ failure lasting >48 h (16).

Recurrence was defined as a new episode of acute pancreatitis meeting standard diagnostic criteria and occurring ≥3 months after the index attack, following complete clinical resolution with a symptom-free interval, to minimize misclassification of early re-admissions attributable to persistent inflammation or local complications of the index episode (17). All recurrent episodes were identified according to clinical diagnoses documented in the medical records and verified by corresponding imaging examinations. Metabolic comorbidities included diabetes, hypertension, fatty liver, and gout. Obesity was defined by a body mass index (BMI) of ≥28.0 kg/m^2^. Lipid-lowering therapy was defined as continuous use of statins, fibrates, or niacin for at least 1 month before admission.

### 2.3 Data collection

Data were retrospectively extracted from the electronic medical record systems of all centers, covering demographic information at admission (age, gender, BMI, marital status, education level), lifestyle habits (smoking and drinking history), comorbidities (diabetes, hypertension, gout, and fatty liver), and history of lipid-lowering drug use. Disease-related data were also recorded, including previous episodes, severity classification of the current episode, and laboratory findings within 24 h of admission, such as routine complete blood count, hepatic and renal function tests, electrolytes, total cholesterol, triglycerides, fasting blood glucose (FBG), LDL-C, HDL-C, C-reactive protein (CRP), and procalcitonin (PCT). To ensure data accuracy, all information was independently extracted and cross-checked by two trained investigators using a standardized data collection form. Any discrepancies were adjudicated by a third researcher.

### 2.4 Lipid assessment, inter-center comparability, and sample quality handling

Admission lipid profiles (triglycerides, total cholesterol, LDL-C, and HDL-C) were extracted from the laboratory information systems of each participating center; only laboratory-validated results released in the system were used for analysis. Blood sampling was performed within 24 h of admission as part of routine clinical care. Lipid sampling was obtained when patients met each center’s pre-analytical requirements for lipid testing; fasting status was assessed by diet history at presentation. Serum lipids were measured in each center’s central laboratory using enzymatic assays on automated chemistry analyzers, and HDL-C was determined using a direct homogeneous method according to manufacturers’ instructions and local standard operating procedures.

Inter-center comparability was supported by national clinical laboratory quality management requirements, including routine internal quality control and participation in applicable external quality assurance programs. Lipid values were harmonized for analysis using uniform units. Specimens with severe lipemia or hemolysis were flagged by the laboratory; results deemed analytically unreliable were not released as valid results and were therefore excluded from analysis. When repeat sampling and re-testing were performed as part of routine clinical care after specimen rejection, the subsequent validated result was used when available.

### 2.5 Follow-up and study endpoints

Follow-up data were obtained through outpatient visits, review of inpatient medical records, and structured telephone interviews. Trained nurses conducted telephone follow-ups at 1, 3, and 6 months after discharge; recurrence at 12 months and longer-term outcomes were ascertained via ongoing outpatient follow-up and supplemented by medical record review. For suspected recurrences managed outside participating centers, available medical documentation (e.g., discharge summaries, laboratory results, and imaging reports) was retrieved and each episode was adjudicated against standard diagnostic criteria before confirmation. The primary endpoint was overall post-discharge recurrence of HTG-AP, with secondary endpoints of recurrence within 6 and 12 months. Participants without recurrence were censored at the last successful follow-up or the study end date (June 30, 2025), whichever occurred first; patients with missing follow-up information were excluded (*n* = 9; Figure 1).

### 2.6 Statistical analysis

Continuous variables were summarized as median (interquartile range, IQR) and compared using the Mann–Whitney U test; categorical variables were presented as *n* (%) and compared using the chi-square or Fisher’s exact test, as appropriate. Recurrence risk was analyzed using Cox proportional hazards models and reported as hazard ratios (HRs) with 95% confidence intervals (CIs). HDL-C was modelled both continuously and by tertiles. Four hierarchical models were prespecified: Model 1, unadjusted; Model 2, adjusted for gender, age, and BMI; Model 3, additionally adjusted for smoking, drinking, metabolic comorbidities, and first episode status; and Model 4, further adjusted for lipid-lowering therapy, admission triglycerides, admission CRP, and acute pancreatitis severity. Kaplan–Meier curves were compared using the log-rank test.

To explore time-dependent effects, Landmark analyses were performed for recurrence risk at 6 and 12 months, with separate Cox models constructed for each time window. An interaction term between HDL-C and log(time) was incorporated to assess the proportional hazards assumption, with adjustment for Model 3 covariates and lipid-lowering therapy use. The restricted mean recurrence-free survival time (RMST) within 12 months was used to quantify absolute risk differences. The dose–response relationship was evaluated using a restricted cubic spline (RCS) model adjusted according to Model 4. Subgroup analyses were stratified by gender, obesity, first onset, and metabolic comorbidities to test for interactions, and joint effects were assessed on an additive scale. Multiple recurrence events were analyzed using negative binomial regression to estimate incidence rate ratios (IRRs).

A Bayesian Cox sensitivity analysis was performed by jointly including admission triglycerides, total cholesterol, LDL-C, and HDL-C (each per 1-SD increase) with the fully adjusted covariates and applying weakly informative regularizing priors. Results are reported as posterior median HRs with 95% credible intervals (CrIs) and posterior probabilities, and convergence was assessed using Rhat and effective sample size.

To reduce confounding, inverse probability of treatment weighting (IPTW) based on propensity scores was applied, and the main analyses were repeated in the weighted cohort. HDL-C was dichotomised using the T3 cut-point (high HDL-C: ≥T3; low HDL-C: <T3). Propensity scores (PS) for low HDL-C were estimated with multivariable logistic regression including prespecified baseline covariates: gender, age, BMI, education level, marital status, smoking history, drinking history, diabetes, hypertension, gout, hepatic steatosis, lipid-lowering agent use, and first episode status. IPTW weights were defined as 1/PS for the low HDL-C group and 1/(1 − PS) for the high HDL-C group, with extreme weights truncated at the 1st and 99th percentiles to improve stability. Covariate balance was assessed using standardized mean differences (SMDs). Weighted Cox models were fitted using robust sandwich variance estimators. All statistical analyses and visualizations were performed using R version 4.4.3 and Python version 3.12.3, with a two-sided *p* value <0.05 considered statistically significant.

## 3 Results

### 3.1 Baseline characteristics

In total, 440 patients diagnosed with HTG-AP were enrolled, with a median age of 39.00 (33.00–46.25) years. Among them, 80.91% (356/440) were male, with the median follow-up period was 14.0 (8.0–26.0) months. Participants were categorized into three groups based on HDL-C levels at admission, and comparisons were made across groups. The results showed that patients in the T1 group had the highest recurrence rate (46.90%), tended to be younger, and had higher proportions of prior episodes and severe cases. Compared with the T2 and T3 groups, patients in the T1 group had significantly higher levels of FBG, triglycerides, and total cholesterol, indicating a heavier metabolic burden and less frequent use of lipid-lowering therapy (Table 1).

**Table 1:** Baseline characteristics and recurrence by tertiles of admission HDL-C.

Variables	HDL-C			*p*
	T1 (*n* = 145) <0.49	T2 (*n* = 148) 0.49–0.72	T3 (*n* = 147) ≥0.72	
Gender, *n* (%)				0.349
Male	112 (77.24)	124 (83.78)	120 (81.63)	
Female	33 (22.76)	24 (16.22)	27 (18.37)	
Age, years	37.00 (32.00,45.00)	38.50 (33.00,46.25)	40.00 (34.00,47.00)	0.020
BMI, kg/m2	26.57 (24.56,29.30)	27.22 (25.24,29.76)	26.35 (24.02,28.57)	0.020
Education level, *n* (%)				0.335
Primary school or below	27 (18.62)	22 (14.86)	19 (12.93)	
Middle school	59 (40.69)	51 (34.46)	47 (31.97)	
High school	25 (17.24)	28 (18.92)	33 (22.45)	
Bachelor’s degree or above	34 (23.45)	47 (31.76)	48 (32.65)	
Smoking history, *n* (%)				0.352
Never smoked	78 (53.79)	88 (59.46)	84 (57.14)	
Current smoker	65 (44.83)	54 (36.49)	61 (41.50)	
Former smoker	2 (1.38)	6 (4.05)	2 (1.36)	
Drinking history, *n* (%)				0.071
Never drinker	81 (55.86)	95 (64.19)	80 (54.42)	
Current drinker	60 (41.38)	50 (33.78)	67 (45.58)	
Former drinker	4 (2.76)	3 (2.03)	0 (0.00)	
Diabetes, *n* (%)				0.181
No	86 (59.31)	80 (54.05)	95 (64.63)	
Yes	59 (40.69)	68 (45.95)	52 (35.37)	
Hypertension, *n* (%)				0.058
No	123 (84.83)	117 (79.05)	108 (73.47)	
Yes	22 (15.17)	31 (20.95)	39 (26.53)	
Gout, *n* (%)				0.226
No	138 (95.17)	133 (89.86)	136 (92.52)	
Yes	7 (4.83)	15 (10.14)	11 (7.48)	
Hepatic steatosis, *n* (%)				0.300
No	27 (18.62)	25 (16.89)	35 (23.81)	
Yes	118 (81.38)	123 (83.11)	112 (76.19)	
Lipid-lowering agents, *n* (%)				<0.001
No	122 (84.14)	102 (68.92)	96 (65.31)	
Yes	23 (15.86)	46 (31.08)	51 (34.69)	
Severity of pancreatitis, *n* (%)				0.006
Mild	66 (45.52)	81 (54.73)	97 (65.99)	
Moderate	38 (26.21)	39 (26.35)	29 (19.73)	
Severe	41 (28.28)	28 (18.92)	21 (14.29)	
First episode, *n* (%)				0.032
No	50 (34.48)	46 (31.08)	31 (21.09)	
Yes	95 (65.52)	102 (68.92)	116 (78.91)	
WBC (×10^9^)	12.81 (9.75,15.96)	12.36 (9.51,15.50)	11.94 (9.84,14.86)	0.290
Hemoglobin (g/L)	157.00 (140.00,177.00)	151.00 (135.00,164.00)	149.00 (136.00,158.00)	0.001
PLT (×10^9^)	231.00 (186.00,271.00)	229.00 (180.00,279.00)	222.00 (194.00,270.00)	0.970
ALT (U/L)	30.00 (21.00,52.00)	28.25 (19.00,46.47)	29.00 (20.00,43.70)	0.518
AST (U/L)	31.00 (20.20,49.00)	25.10 (20.00,35.92)	23.00 (16.75,36.00)	<0.001
ALB (g/L)	39.10 (35.70,43.40)	42.00 (37.77,45.37)	42.30 (38.00,44.90)	0.002
Cr (μmol/L)	61.00 (49.60,78.10)	69.30 (59.51,84.25)	70.00 (55.70,81.50)	0.008
BUN (mmol/L)	3.98 (3.10,5.00)	4.10 (3.20,5.10)	3.80 (2.85,4.85)	0.196
Serum amylase (U/L)	61.00 (49.60,78.10)	69.30 (59.51,84.25)	70.00 (55.70,81.50)	0.008
Serum calcium (mmol/L)	2.08 (1.87,2.24)	2.17 (2.00,2.26)	2.20 (2.06,2.31)	<0.001
PCT (ng/mL)	0.26 (0.09,1.34)	0.21 (0.07,0.66)	0.15 (0.06,0.51)	0.008
CRP (mg/L)	119.80 (44.40,189.00)	104.45 (21.17,193.00)	110.00 (35.65,189.95)	0.629
FBG (mmol/L)	10.30 (6.90,12.85)	9.50 (7.18,12.35)	8.20 (6.25,11.80)	0.003
Total cholesterol (mmol/L)	11.70 (8.27,16.26)	8.72 (6.20,11.59)	6.80 (5.37,9.01)	<0.001
Triglycerides (mmol/L)	22.84 (16.96,37.10)	15.95 (8.56,22.95)	7.53 (4.35,14.61)	<0.001
LDL-C (mmol/L)	2.80 (1.40,4.43)	2.19 (1.20,3.25)	2.84 (1.71,4.12)	0.002
Post-discharge recurrence, *n* (%)				<0.001
No	309 (70.23)	309 (70.23)	309 (70.23)	
Yes	68 (46.90)	37 (25.00)	26 (17.69)	

WBC, white blood cell count; PLT, platelet count; ALT, alanine aminotransferase; AST, aspartate aminotransferase; ALB, albumin; Cr, creatinine; BUN, blood urea nitrogen; PCT, procalcitonin; CRP, C-reactive protein; FBG, fasting blood glucose; LDL-C, low-density lipoprotein cholesterol.

### 3.2 Admission HDL-C and recurrence risk

**Figure 2:**
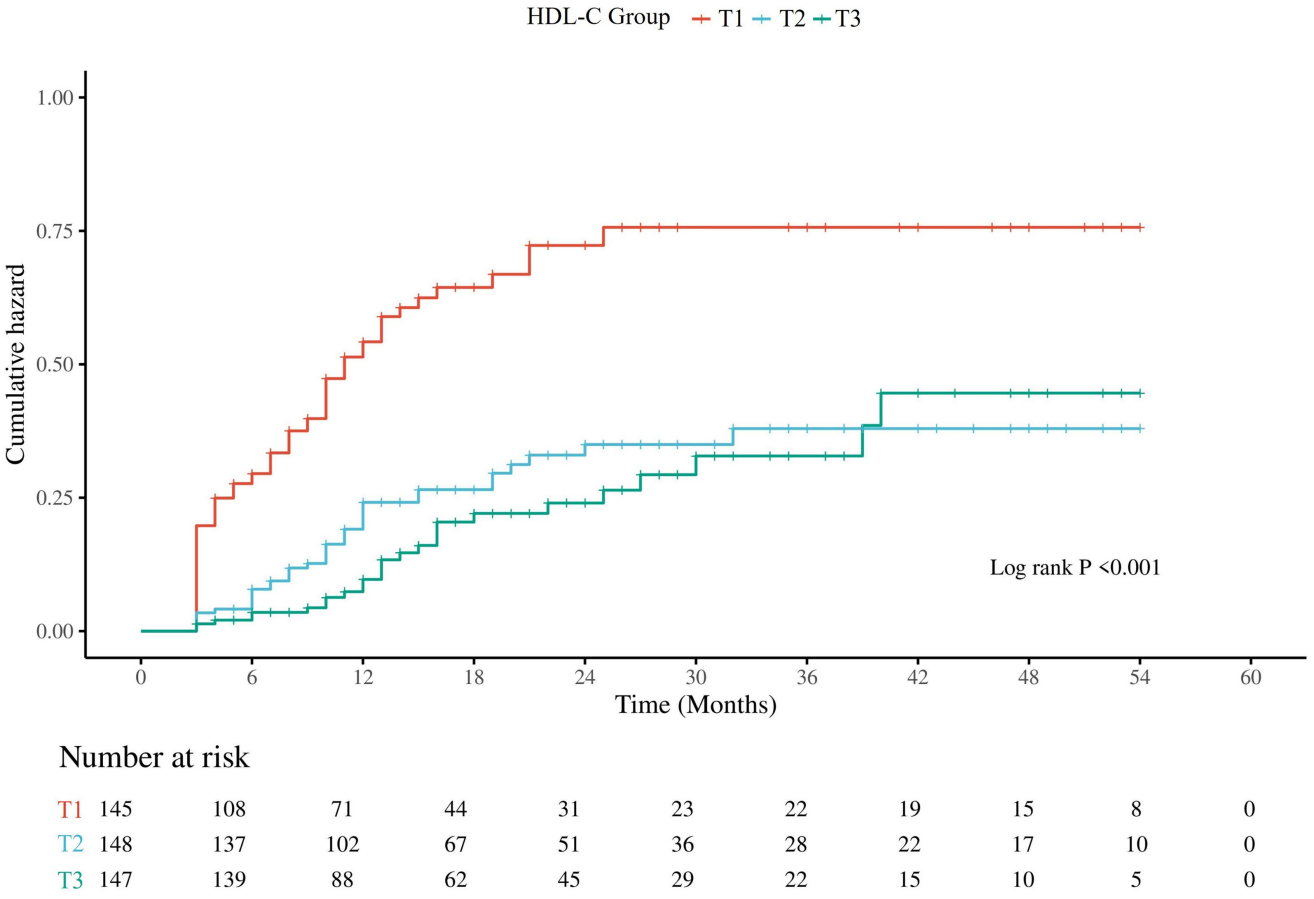
Cumulative hazard of recurrence by admission HDL-C tertiles.

Multivariate Cox regression demonstrated a strong negative correlation between HDL-C levels at admission and the overall recurrence risk of HTG-AP. In the fully adjusted model, each 1 mmol/L increase in HDL-C was associated with a 77% reduction in recurrence risk (HR = 0.23, 95% CI: 0.11–0.48, *p* < 0.001). Compared with the T1 group, recurrence risk decreased by 58% in the T2 group (HR = 0.42, 95% CI: 0.27–0.64) and by 67% in the T3 group (HR = 0.33, 95% CI: 0.19–0.55), with similar protective associations observed for recurrence within 6 and 12 months post-discharge (Table 2; Supplementary Table 1). Kaplan–Meier curves further demonstrated that patients in the T1 group consistently exhibited the highest recurrence risk, which increased rapidly during the early post-discharge period (Log-rank *p* < 0.001) (Figure 2). Furthermore, negative binomial regression showed that the recurrence risk in the T1 group was 79% higher than that in the T3 group (IRR = 1.79, 95% CI: 1.06–3.02) (Supplementary Table 2), further supporting the inverse association between HDL-C and recurrence risk.

In a sensitivity analysis to assess the robustness of our findings and to address potential collinearity among admission lipid measures, we fitted a Bayesian Cox proportional hazards model simultaneously including admission triglycerides, total cholesterol, LDL-C, and HDL-C (each standardized per 1-SD increase). The inverse association between HDL-C and recurrence risk remained robust (median HR 0.613, 95% credible interval 0.485–0.771; posterior probability that HR < 1 was 1.00) (Supplementary Table 3; Supplementary Figure 1).

**Table 2:** Cox regression analysis of admission HDL-C and overall recurrence risk in patients with HTG-AP.

Variables	Model1		Model2		Model3		Model4	
	HR (95%CI)	*p*	HR (95%CI)	*p*	HR (95%CI)	*p*	HR (95%CI)	*p*
HDL-C	0.22 (0.11 ~ 0.42)	<0.001	0.22 (0.11 ~ 0.43)	<0.001	0.23 (0.12 ~ 0.46)	<0.001	0.23 (0.11 ~ 0.48)	<0.001
T1	Reference		Reference		Reference		Reference	
T2	0.42 (0.28 ~ 0.63)	<0.001	0.43 (0.29 ~ 0.64)	<0.001	0.42 (0.28 ~ 0.63)	<0.001	0.42 (0.27 ~ 0.64)	<0.001
T3	0.32 (0.20 ~ 0.50)	<0.001	0.33 (0.21 ~ 0.52)	<0.001	0.34 (0.22 ~ 0.55)	<0.001	0.33 (0.19 ~ 0.55)	<0.001
*p* for trend	<0.001		<0.001		<0.001		<0.001	

HR, hazard ratio; CI, confidence interval.

Model1: Crude.

Model2: Adjust: Gender, Age, BMI.

Model3: Adjust: gender, age, BMI, smoking history, drinking history, diabetes, hypertension, gout, hepatic steatosis, first episode.

Model4: Adjust: gender, age, BMI, smoking history, drinking history, diabetes, hypertension, gout, hepatic steatosis, first episode, lipid-lowering agents, severity of pancreatitis, CRP, triglycerides.

### 3.3 Dose–response relationship

**Figure 3:**
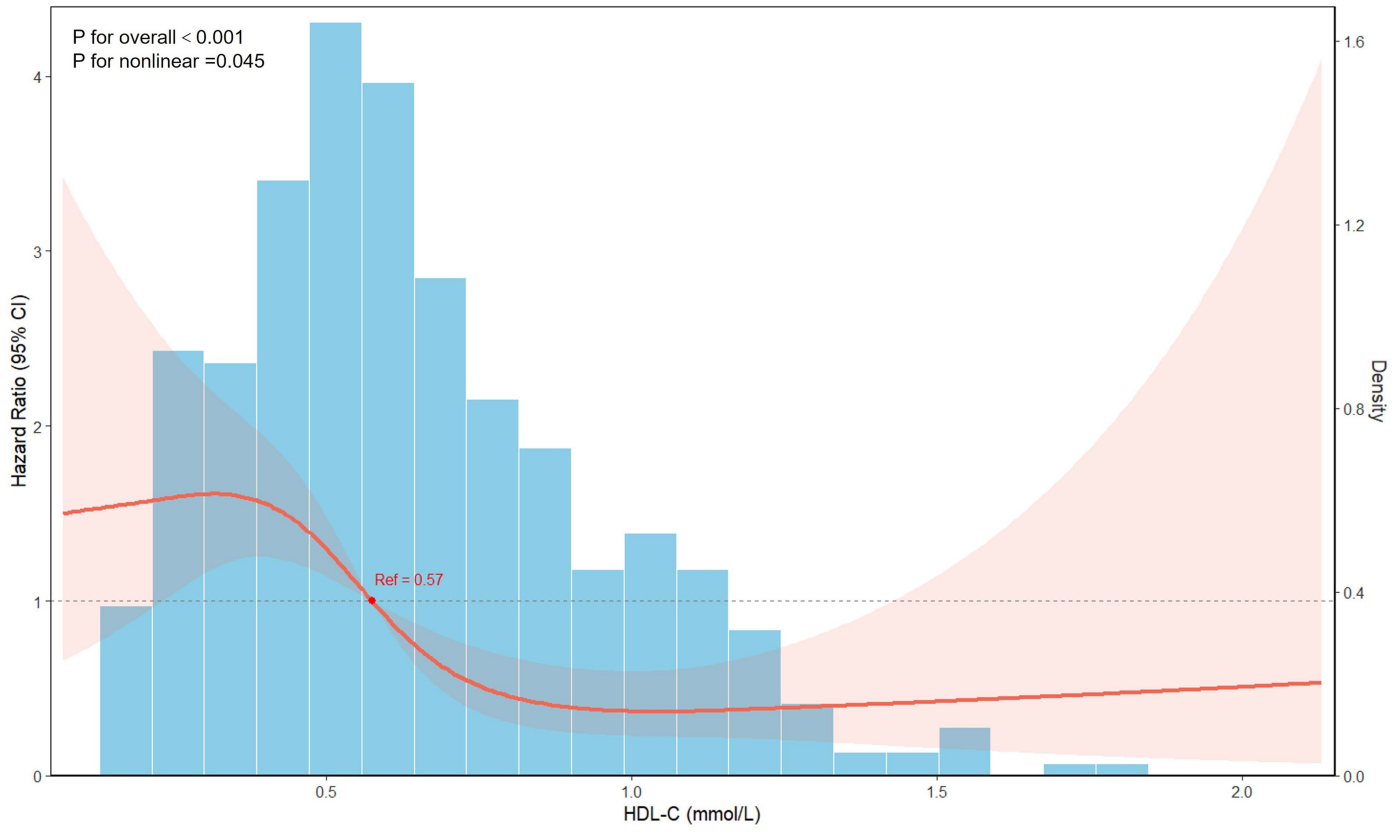
Restricted cubic spline analysis of the association between admission HDL-C and recurrence risk.

On this basis, a RCS analysis was performed to evaluate the dose–response relationship between HDL-C levels and recurrence risk (Figure 3). After adjusting for covariates in Model 4, low HDL-C levels were significantly associated with an increased recurrence risk, which progressively decreased with rising HDL-C and plateaued at higher concentrations.

### 3.4 Time-dependent effects of HDL-C

**Figure 4:**
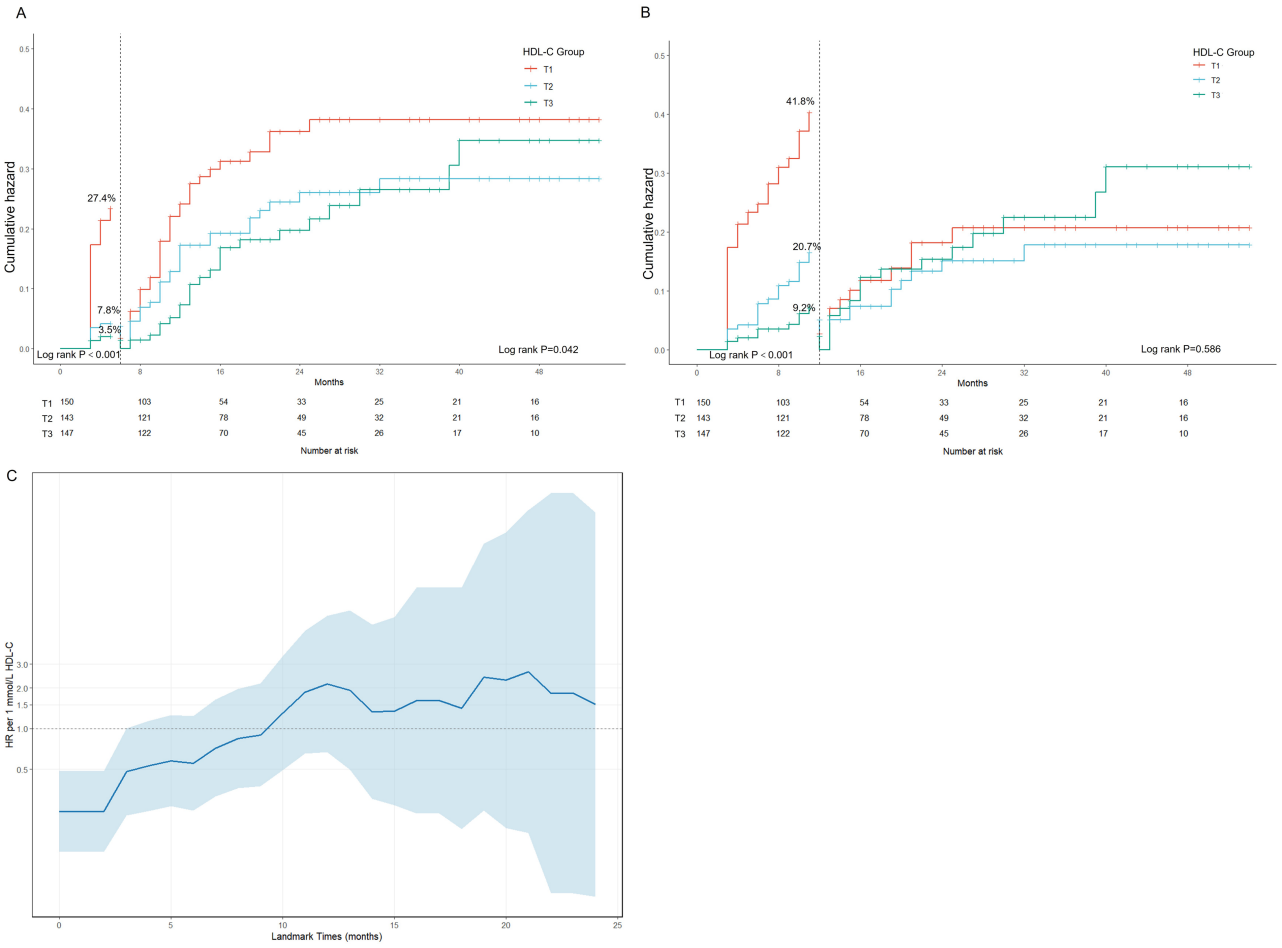
Landmark and time-dependent analysis of HDL-C and recurrence risk. **(A)** Landmark analysis for 6 months; **(B)** landmark analysis for 12 months; **(C)** time-dependent effect analysis.

To delineate the distinct effects of HDL-C on early versus late recurrence, a landmark analysis was conducted (Figure 4; Supplementary Table 4). The Kaplan–Meier curves indicated that the predictive effect of HDL-C was primarily concentrated within the first 12 months after discharge. Patients with low HDL-C levels had a higher recurrence rate within 0–6 months, with rates of 27.4% in the T1 group versus 7.8 and 5.1% in the T2 and T3 groups, respectively (log-rank *p* < 0.001). The difference remained significant at 12 months post-discharge (log-rank *p* = 0.042), but after 12 months, the survival curves for all groups converged progressively (log-rank *p* = 0.568).

The Cox regression results from the landmark analysis showed a consistent trend (Table 3). Within 6 months after discharge, each 1 mmol/L increase in HDL-C was associated with a 95.5% reduction in recurrence risk (HR = 0.045, 95% CI: 0.012–0.171, *p* < 0.001). During the 6–12 month period, this protective effect remained significant (HR = 0.176, 95% CI: 0.054–0.568, *p* = 0.004). However, beyond 12 months, the association between HDL-C and recurrence risk was no longer significant (*p* = 0.197). According to the RMST analysis (Supplementary Table 4), within 12 months, the mean recurrence-free survival time in T1 and T2 groups was 2.2 months (p < 0.001) and 0.5 months (*p* = 0.026) shorter than that of the T3 group, respectively. The time-dependent analysis also demonstrated that the protective effect of HDL-C was mainly concentrated in the early post-discharge period and gradually diminished over time.

**Table 3:** Landmark Cox regression and time-dependent effect of admission HDL-C on recurrence risk.

Model	Sample size	Events	HR (per 1 mmol/L)	95% CI	*p*
0–6 months	440	53	0.045	0.012 ~ 0.171	<0.001
6–12 months	367	45	0.176	0.054 ~ 0.568	0.004
>12 months	242	33	2.148	0.672 ~ 6.868	0.197
Time-dependent effect	440	131	0.005	0.001 ~ 0.045	<0.001
HDL × log(time)	-	-	5.899	2.402 ~ 14.487	<0.001

### 3.5 Subgroup and interaction analyses

**Figure 5:**
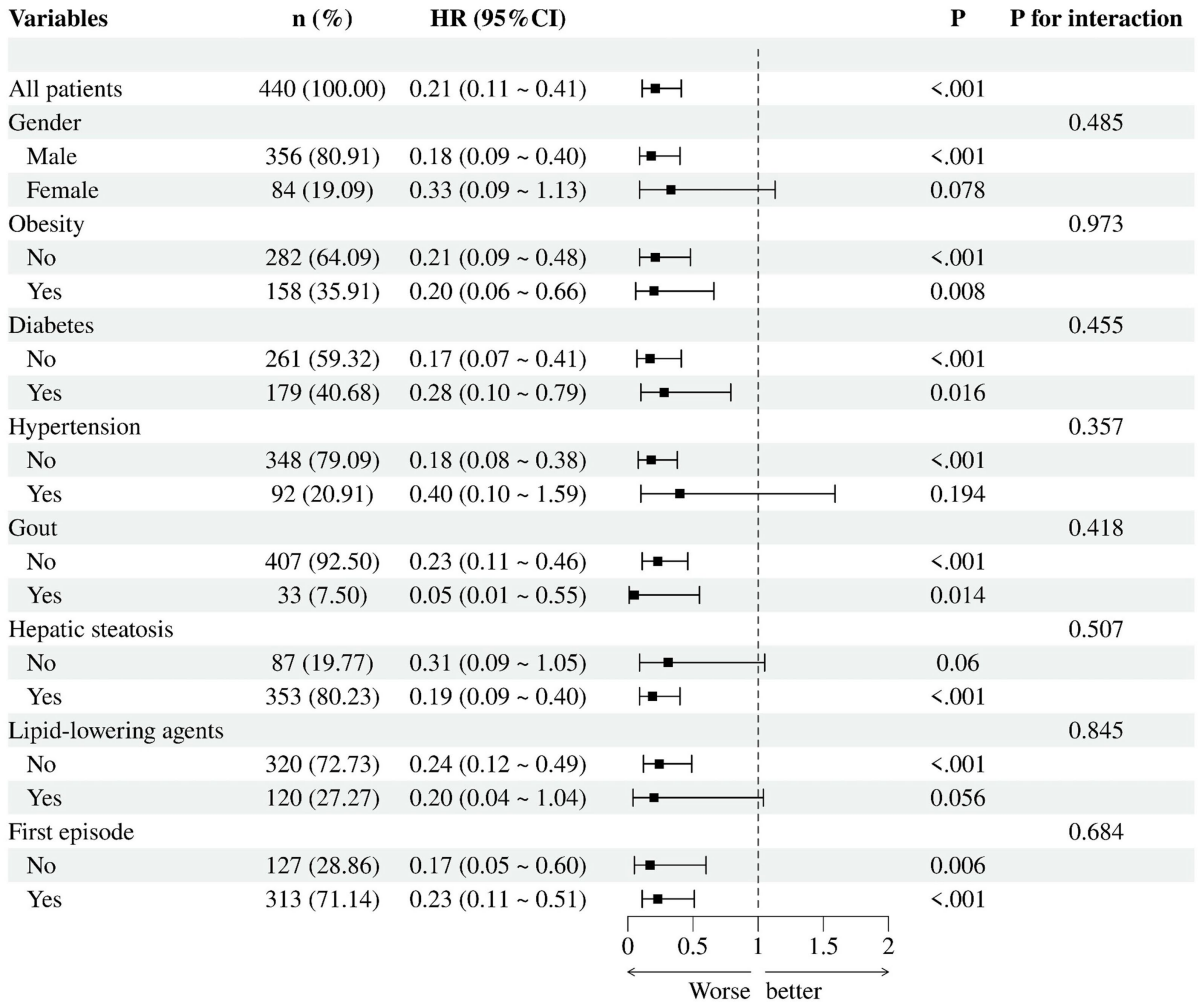
Subgroup analysis of admission HDL-C and recurrence risk.

Subgroup analyses (Figure 5) showed that lower admission HDL-C levels were consistently associated with a higher recurrence risk across the examined strata, although the magnitude of the association varied across subgroups. However, multiplicative interaction tests provided no evidence of effect modification by gender, obesity, diabetes, hypertension, gout, fatty liver, lipid-lowering drug use, or first-onset status (all *p* for interaction > 0.05). In the additive interaction analysis (Supplementary Table 5), the joint exposure of low HDL-C and metabolic comorbidities was associated with a higher recurrence risk compared with the reference group, but the relative excess risk due to interaction (RERI) was not statistically significant, indicating no clear evidence of additive interaction.

### 3.6 IPTW-adjusted analysis

To verify the robustness of the results, we repeated the primary analyses using IPTW. HDL-C levels at admission were divided into high and low groups using the T3 tertile as the cutoff. Before weighting, differences existed between the two groups in age, BMI, marital status, lipid-lowering agents use, and first-onset proportion; after IPTW adjustment, baseline covariates were well balanced and the group distributions were largely comparable (Supplementary Figure 2; Table 4).

**Table 4:** Distribution of propensity scores before and after IPTW.

Variable	Before IPTW				After IPTW			
	High HDL-C (*n* = 142)	Low HDL-C (*n* = 298)	*p*	SMD	High HDL-C (*n* = 426.9)	Low HDL-C (*n* = 433.8)	*p*	SMD
Age, years	40.00 (34.00, 47.00)	38.00 (32.00, 46.00)	0.026	0.261	39.00 (34.00, 46.00)	38.00 (33.00, 47.00)	0.763	0.035
BMI, kg/m^2^	26.35 (24.06, 28.51)	26.95 (24.97, 29.41)	0.025	0.260	26.64 (24.28, 28.69)	26.74 (24.75, 29.29)	0.563	0.095
Gender, *n* (%)								
Male	115 (80.99)	241 (80.87)	>0.999	0.003	335.9 (78.68)	350.1 (80.70)	0.662	0.050
Female	27 (19.01)	57(19.13)			91.0 (21.32)	83.7 (19.30)		
Education level, *n* (%)								
Primary school or below	18 (12.68)	50 (16.78)	0.263	0.205	62.0 (14.52)	66.4 (15.31)	0.989	0.039
Middle school	45 (31.69)	112 (37.58)			159.7 (37.41)	155.2 (35.78)		
High school	31 (21.83)	55 (18.46)			84.8 (19.87)	85.5 (19.71)		
Bachelor’s degree or above	48 (33.80)	81 (27.18)			120.4 (28.20)	126.7 (29.20)		
Marital status, *n* (%)								
Single	18 (12.68)	39 (13.09)	0.002	0.297	51.5 (12.06)	58.1 (13.39)	0.165	0.172
Married	118 (83.10)	259 (86.91)			369.4 (86.53)	375.7 (86.61)		
Divorced	6 (4.22)	0 (0.00)			6.0 (1.41)	0.0 (0.00)		
Smoking history, *n* (%)								
Never smoked	82 (57.75)	168 (56.38)	0.313	0.172	252.0 (59.03)	246.7 (56.87)	0.926	0.047
Current smoker	59 (41.55)	121 (40.60)			166.5 (39.00)	177.0 (40.80)		
Former smoker	1 (0.70)	9 (3.02)			8.4 (1.97)	10.0 (2.31)		
Drinking history, *n* (%)								
Never drinker	78 (54.93)	178 (59.73)	0.085	0.254	258.3 (60.51)	253.2 (58.37)	0.147	0.183
Current drinker	64 (45.07)	113 (37.92)			168.6 (39.49)	173.6 (40.80)		
Former drinker	0 (0.0)	7 (2.35)			0.0 (0.00)	7.0 (1.61)		
Diabetes, *n* (%)								
No	92 (64.79)	169 (56.71)	0.131	0.166	259.3 (60.74)	256.2 (59.06)	0.761	0.034
Yes	50 (35.21)	129 (43.29)			167.6 (39.26)	177.6 (40.94)		
Hypertension, *n* (%)								
No	104 (73.24)	244 (81.88)	0.050	0.208	340.4 (79.74)	342.6 (78.98)	0.860	0.018
Yes	38 (26.76)	54 (18.12)			86.5 (20.26)	91.2 (21.02)		
Gout, *n* (%)								
No	131 (92.25)	276 (92.62)	>0.999	0.014	393.5 (92.18)	400.7 (92.37)	0.949	0.007
Yes	11 (7.75)	22 (7.38)			33.4 (7.82)	33.1 (7.63)		
Hepatic steatosis, *n* (%)								
No	35 (24.65)	52 (17.45)	0.100	0.177	92.3 (21.62)	85.5 (19.71)	0.663	0.047
Yes	107 (75.35)	246 (82.55)			334.6 (78.38)	348.3 (80.29)		
Lipid-lowering agents, *n* (%)								
No	93 (65.49)	227 (76.17)	0.025	0.237	311.9 (73.06)	314.7 (72.55)	0.912	0.012
Yes	49 (34.51)	71 (23.83)			115.0 (26.94)	119.1 (27.45)		
First episode, *n* (%)								
No	31 (21.83)	96 (32.21)	0.033	0.235	105.8 (24.78)	124.8 (28.77)	0.423	0.091
Yes	111 (78.17)	202 (67.79)			321.1 (75.22)	309.0 (71.23)		

**Figure 6:**
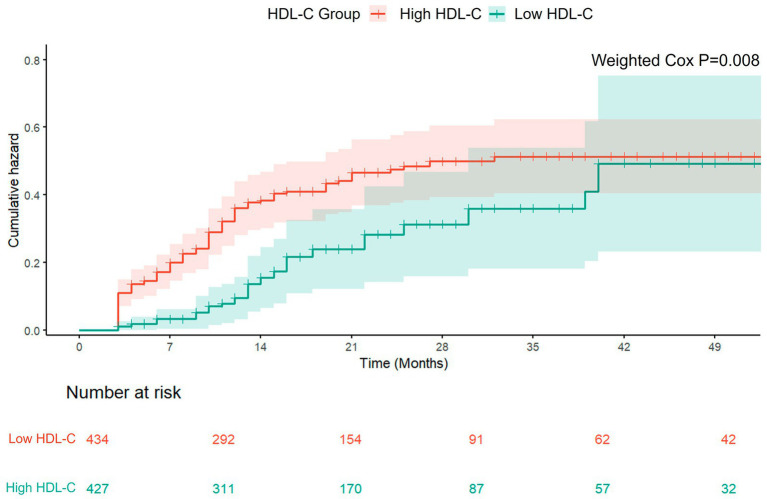
IPTW-adjusted cumulative hazard of recurrence by HDL-C level.

After weighting, Cox regression analyses revealed that patients with low HDL-C consistently exhibited a higher recurrence risk than those with high HDL-C at all follow-up time windows (Figure 6; Table 5). Within the first 0–6 months after discharge, the low HDL-C group had a 76% higher recurrence risk (aHR = 1.76, 95% CI: 1.12–2.77, *p* = 0.014). During the 6–12 month period, the risk further increased to 127% (aHR = 2.27, 95% CI: 1.38–3.73, *p* = 0.001). This difference was most pronounced during the first year of follow-up (aHR = 3.79, 95% CI: 1.92–7.48, *p* < 0.001). During extended follow-up, the risk difference became less pronounced, yet the overall recurrence risk remained significantly elevated among patients with low HDL-C (aHR = 1.80, 95% CI: 1.14–2.83, *p* = 0.011).

**Table 5:** IPTW-adjusted Cox regression for recurrence risk by HDL-C level.

HDL-C level	6-month recurrence		6-12 month recurrence		12-month recurrence		Overall recurrence	
	aHR (95%CI)	*p*	aHR (95%CI)	*p*	aHR (95%CI)	*p*	aHR (95%CI)	*p*
High HDL-C	Reference		Reference		Reference		Reference	
Low HDL-C	1.76 (1.12 ~ 2.77)	0.014	2.27 (1.38 ~ 3.73)	0.001	3.79 (1.92 ~ 7.48)	<0.001	1.80 (1.14 ~ 2.83)	0.011

## 4 Discussion

In this multicenter retrospective cohort study, we systematically investigated the relationship between high-density lipoprotein cholesterol levels at admission and the risk of post-discharge recurrence in patients with hypertriglyceridemia-induced acute pancreatitis. Results indicated that lower admission HDL-C levels were associated with a linear increase in overall recurrence rate, and recurrence risk rose markedly with decreasing HDL-C. Restricted cubic splines analysis suggested a clear dose–response relationship between HDL-C levels and recurrence risk. Multivariate Cox regression analysis further verified that lower HDL-C remained associated with recurrence after multivariable adjustment, with each 1 mmol/L increase in HDL-C associated with an approximately 77% lower hazard of recurrence. Landmark analysis indicated that the association between admission HDL-C and recurrence risk was mainly concentrated within 12 months after discharge; patients with low HDL-C had significantly higher recurrence rates at 6 and 12 months compared with the moderate-to-high HDL-C groups, while the difference gradually diminished beyond 12 months. The IPTW-weighted analysis yielded results consistent with the main analysis, strengthening the robustness of the observed association.

Our findings revealed that HTG-AP patients commonly displayed a distinct metabolic pattern characterized by decreased HDL-C levels during the acute episode, aligning with prior studies (13, 18, 19). HDL-C is not only a key indicator of lipid metabolism but also plays important roles in reverse cholesterol transport, modulation of inflammation, antioxidative activity, immune regulation, and maintenance of endothelial homeostasis (10, 20). Experimental and clinical evidence suggests that HDL-C facilitates the clearance of free fatty acids, thereby reducing lipotoxic injury and attenuating systemic inflammation (21, 22). When HDL-C levels decline, these protective functions may be weakened, potentially contributing to persistent lipotoxicity and inflammation in the setting of hypertriglyceridemia (10). Inflammation can also alter the protein and lipid composition of high-density lipoprotein cholesterol, diminishing its anti-inflammatory and antioxidant capacity and leading to dysfunctional high-density lipoprotein cholesterol formation (23, 24), which increases susceptibility to recurrence during renewed metabolic or inflammatory stress. Moreover, HTG-AP often coexists with metabolic syndrome, diabetes, and obesity. These conditions can further impair high-density lipoprotein cholesterol function and diminish its protective capacity, thereby increasing recurrence risk (5, 13, 25–27).

Previous studies have mostly focused on the role of high-density lipoprotein cholesterol in the severity and short-term mortality of acute pancreatitis, suggesting that lower HDL-C levels reflect heightened inflammatory activity and reduced antioxidant capacity, which are associated with organ dysfunction and increased mortality (11–14). However, we found that HDL-C levels are not merely biochemical markers of acute inflammation but are also associated with the tendency for disease recurrence after remission. After adjustment for pancreatitis severity, C-reactive protein, and triglycerides at admission, patients with lower admission HDL-C remained at higher risk of recurrence, suggesting that reduced HDL-C may reflect a persistent metabolic susceptibility rather than merely an acute-phase response. A recent Mendelian randomization study reported that lower HDL-C levels are associated with an increased risk of acute pancreatitis and further suggested that pancreatitis episodes may, in turn, downregulate HDL-C through inflammatory mechanisms (18). This finding provides new insights into the recurrence mechanism of HTG-AP, suggesting that lower HDL-C levels may capture a more persistent metabolic-inflammatory susceptibility beyond the acute-phase response, with implications for inflammatory regulation and lipid homeostasis.

Notably, previous studies have identified low HDL-C as a stable marker of prolonged exposure to elevated triglycerides and residual cholesterol, analogous to the role of HbA1c in reflecting long-term average blood glucose levels (28). In contrast to triglycerides, which are readily influenced by diet, pharmacologic interventions, and inflammatory status, HDL-C levels exhibit greater intraindividual stability, with standardized, convenient assays that make them highly accessible and broadly applicable (29–31). Moreover, high-density lipoprotein cholesterol and triglycerides may have complementary value for risk stratification. Triglycerides reflect the current metabolic load and are strongly influenced by short-term factors, making them suitable for dynamic assessment with repeated measurements over time (32, 33). By contrast, HDL-C may better capture the longer-term metabolic background and susceptibility, which may support earlier identification of individuals at higher risk and inform more individualized follow-up (28). In our analyses, the association between admission HDL-C and recurrence risk was mainly observed within the first 12 months after discharge, suggesting that this period may be an important window for closer monitoring and risk management. Among patients with low HDL-C, more intensive metabolic monitoring and reinforced counselling on lipid control and lifestyle modification may be considered to support post-discharge care and potentially reduce early recurrence risk.

This study has several strengths. First, we used a multicenter retrospective cohort with an adequate sample size, which provides a representative overview of the clinical characteristics and recurrence patterns of patients with HTG-AP. Second, we systematically examined the association between admission HDL-C levels and post-discharge recurrence risk, and we confirmed the consistency of the findings using multiple analytic approaches, including multivariable Cox regression, landmark analysis, and IPTW weighting. These complementary analyses enhance the robustness of our results. Finally, we characterized both the dose–response relationship and the temporal pattern linking HDL-C to recurrence risk, which may help identify patients at higher risk of recurrence.

Limitations warrant consideration. First, given the retrospective design, residual confounding is possible despite comprehensive adjustment and the IPTW analyses. Second, although we captured key baseline metabolic and clinical characteristics at the index admission, post-discharge data were not uniformly available across centers. In particular, longitudinal triglyceride monitoring and detailed information on lifestyle changes (e.g., diet, alcohol intake, weight control, and physical activity) were not systematically recorded or standardized, and adherence to lipid-lowering therapy could not be reliably ascertained. Therefore, we could not quantify how differences in post-discharge management might influence recurrence risk. Third, by requiring an interval of ≥3 months between attacks in accordance with consensus definitions of recurrent acute pancreatitis, we may have missed very early recurrent events; thus, the incidence of recurrence may be slightly underestimated. Finally, as our cohort was derived from a Chinese population, the generalizability of these findings requires validation in other ethnic and geographic settings. Future research should aim to clarify the potential causal relationship and underlying mechanisms linking HDL-C to recurrence risk in HTG-AP through prospective, large-sample, multicenter cohorts, while investigating underlying mechanisms and intervention potential, thereby informing new preventive strategies against recurrence.

## 5 Conclusion

Low HDL-C serves as an independent predictor of HTG-AP recurrence, exhibiting a linear increase in risk, with its prognostic effect most evident during the first 12 months after discharge. High-density lipoprotein cholesterol, being a reliable and readily measurable metabolic indicator, may help identify high-risk individuals and inform strategies for recurrence prevention and long-term management.

## Data Availability

The original contributions presented in the study are included in the article/Supplementary material, further inquiries can be directed to the corresponding authors.
