# Supplementary figures and images for: Admission HDL-C and recurrence risk of hypertriglyceridemia-induced acute pancreatitis: a multicenter cohort study
Source: Front Nutr. 2026 Jan 30;13:1741265. doi: 10.3389/fnut.2026.1741265 (PMC12900692; doi:10.3389/fnut.2026.1741265)

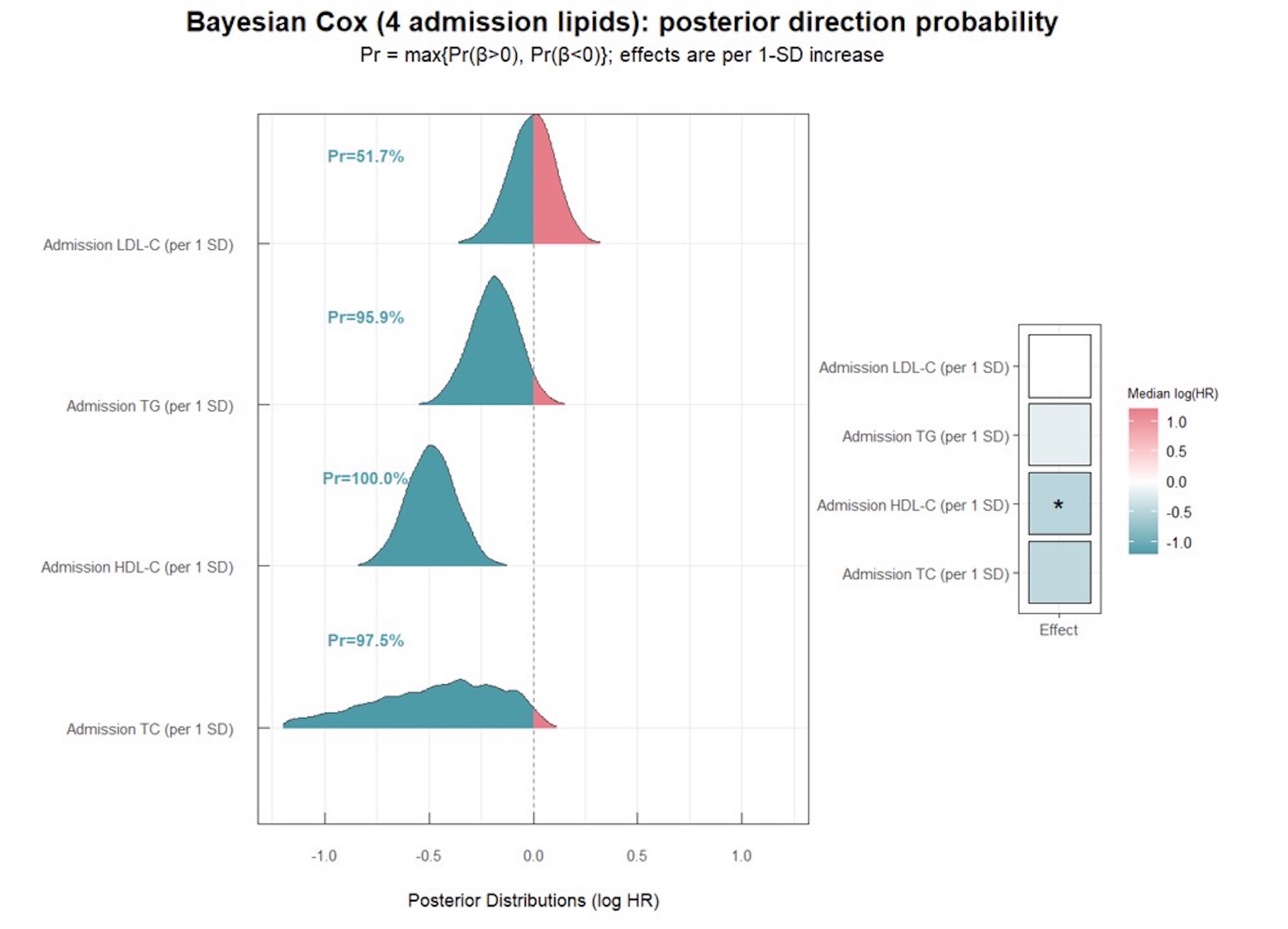

Supplement: Supplementary file 1 [file Image_1.jpeg]

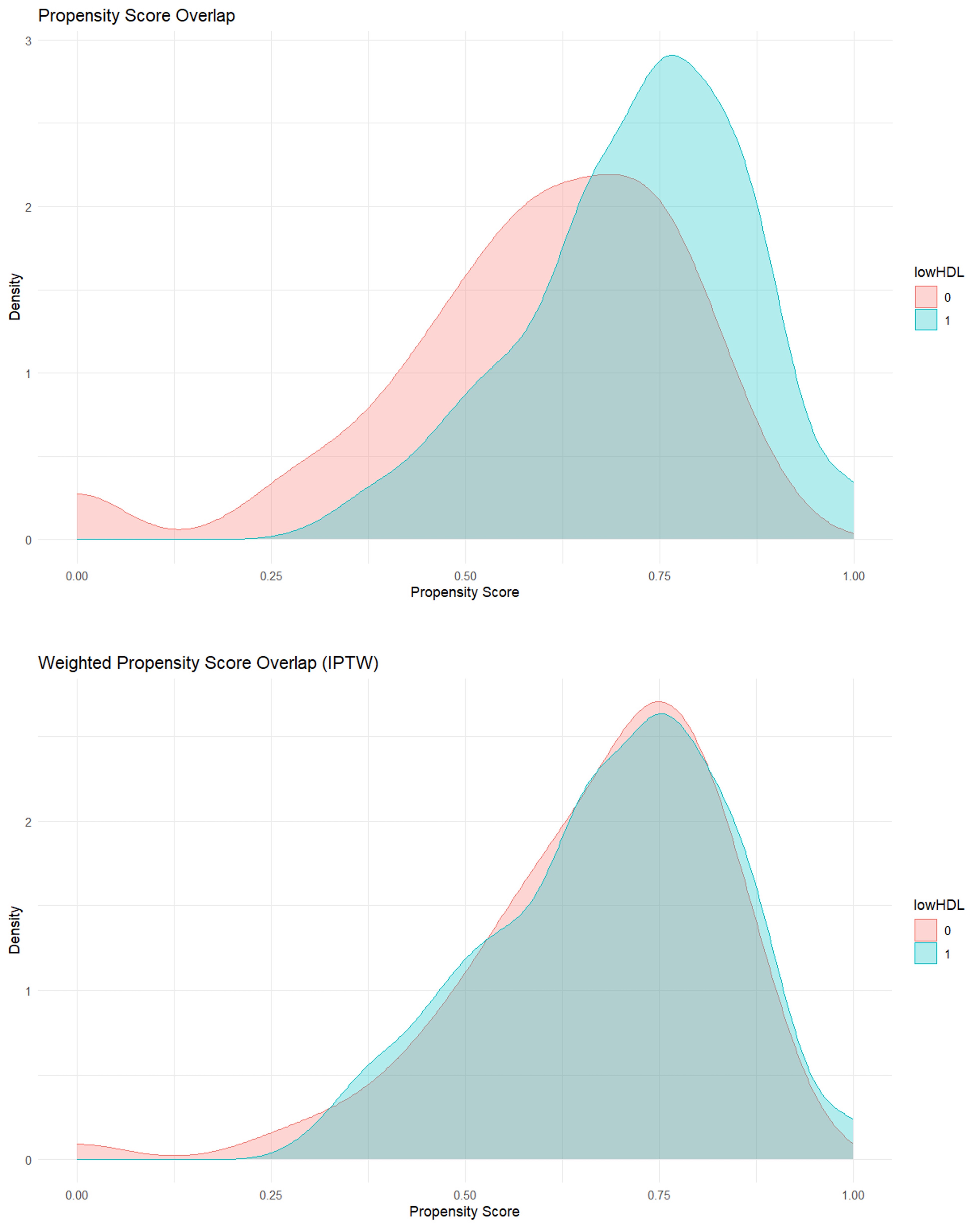

Supplement: Supplementary file 2 [file Image_2.jpeg]
