# Supplementary material for: Admission HDL-C and recurrence risk of hypertriglyceridemia-induced acute pancreatitis: a multicenter cohort study
Source: Front Nutr. 2026 Jan 30;13:1741265. doi: 10.3389/fnut.2026.1741265 (PMC12900692; doi:10.3389/fnut.2026.1741265)
Supplement: Supplementary file 3 [file Table_1.docx]

Supplementary Table 1. Cox regression analysis of admission HDL-C and 6-month and 12-month recurrence risk in patients with HTG-AP

| Variables | Model1 | |  | Model2 | |  | Model3 | |  | Model4 | |
| --- | --- | --- | --- | --- | --- | --- | --- | --- | --- | --- | --- |
|  | HR (95%CI) | *P* |  | HR (95%CI) | *P* |  | HR (95%CI) | *P* |  | HR (95%CI) | *P* |
| **6-month recurrence** | | | | | | | | | | | |
| HDL-C | 0.30 (0.15 ~ 0.59) | <0.001 |  | 0.30 (0.15 ~ 0.60) | <0.001 |  | 0.30 (0.15 ~ 0.59) | <0.001 |  | 0.34 (0.17 ~ 0.69) | 0.003 |
| HDL-C, tertiles | | | | | | | | | | | |
| T1 | Reference |  |  | Reference |  |  | Reference |  |  | Reference |  |
| T2 | 0.60 (0.39 ~ 0.94) | 0.024 |  | 0.61 (0.40 ~ 0.95) | 0.029 |  | 0.60 (0.39 ~ 0.93) | 0.022 |  | 0.62 (0.40 ~ 0.97) | 0.034 |
| T3 | 0.46 (0.29 ~ 0.72) | <0.001 |  | 0.47 (0.30 ~ 0.74) | 0.001 |  | 0.48 (0.30 ~ 0.75) | <0.001 |  | 0.51 (0.32 ~ 0.81) | 0.005 |
| P for trend | <0.001 | |  | <0.001 | |  | <0.001 | |  | <0.001 | |
| **12-month recurrence** | | | | | | | | | | | |
| HDL-C | 0.25 (0.14 ~ 0.45) | <0.001 |  | 0.26 (0.14 ~ 0.46) | <0.001 |  | 0.25 (0.14 ~ 0.46) | <0.001 |  | 0.26 (0.15 ~ 0.50) | <0.001 |
| HDL-C, tertiles | | | | | | | | | | | |
| T1 | Reference |  |  | Reference |  |  | Reference |  |  | Reference |  |
| T2 | 0.61 (0.42 ~ 0.87) | 0.007 |  | 0.61 (0.43 ~ 0.88) | 0.008 |  | 0.59 (0.41 ~ 0.86) | 0.006 |  | 0.61 (0.42 ~ 0.88) | 0.008 |
| T3 | 0.38 (0.26 ~ 0.57) | <0.001 |  | 0.39 (0.26 ~ 0.58) | <0.001 |  | 0.40 (0.27 ~ 0.59) | <0.001 |  | 0.41 (0.27 ~ 0.62) | <0.001 |
| P for trend | <0.001 | |  | <0.001 | |  | <0.001 | |  | <0.001 | |
| HR: Hazard Ratio, CI: Confidence Interval | | | | | | | | | | | |
| Model1: Crude | | | | | | | | | | | |
| Model2: Adjust: Gender, Age, BMI | | | | | | | | | | | |
| Model3: Adjust: Gender, Age, BMI, Smoking history, Drinking history, Diabetes, Hypertension, Gout, Hepatic steatosis, First episode | | | | | | | | | | | |
| Model4: Adjust: Gender, Age, BMI, Smoking history, Drinking history, Diabetes, Hypertension, Gout, Hepatic steatosis, First episode, Lipid-lowering agents, Severity of pancreatitis, CRP, Triglycerides | | | | | | | | | | | |

Supplementary Table 2. Negative binomial regression analysis of admission HDL-C and recurrence incidence

| Variables | Model1 | |  | Model2 | |  | Model3 | |  | Model4 | |
| --- | --- | --- | --- | --- | --- | --- | --- | --- | --- | --- | --- |
|  | IRR (95%CI) | *P* |  | IRR (95%CI) | *P* |  | IRR (95%CI) | *P* |  | IRR (95%CI) | *P* |
| HDL-C | 0.55 (0.33 ~ 0.92) | 0.022 |  | 0.42 (0.22 ~ 0.80) | 0.008 |  | 0.44 (0.23 ~ 0.86) | 0.016 |  | 0.48 (0.21 ~ 1.00) | 0.049 |
| HDL-C, tertiles | | | | | | | | | | | |
| T3 | Reference |  |  | Reference |  |  | Reference |  |  | Reference |  |
| T1 | 1.91 (1.32 ~ 2.79) | <0.001 |  | 2.01 (1.28 ~ 3.14) | 0.002 |  | 1.97 (1.23 ~ 3.14) | 0.005 |  | 1.79 (1.06 ~ 3.02) | 0.028 |
| T2 | 1.27 (0.85 ~ 1.89) | 0.249 |  | 1.65 (1.05 ~ 2.60) | 0.031 |  | 1.62 (1.00 ~ 2.61) | 0.048 |  | 1.55 (0.94 ~ 2.55) | 0.084 |
| P for trend | <0.001 | |  | 0.002 | |  | 0.005 | |  | 0.032 | |
| HR: Hazard Ratio, CI: Confidence Interval | | | | | | | | | | | |
| Model1: Crude | | | | | | | | | | | |
| Model2: Adjust: Gender, Age, BMI | | | | | | | | | | | |
| Model3: Adjust: Gender, Age, BMI, Smoking history, Drinking history, Diabetes, Hypertension, Gout, Hepatic steatosis, First episode | | | | | | | | | | | |
| Model4: Adjust: Gender, Age, BMI, Smoking history, Drinking history, Diabetes, Hypertension, Gout, Hepatic steatosis, First episode, Lipid-lowering agents, Severity of pancreatitis, CRP, Triglycerides | | | | | | | | | | | |

Supplementary Table 3. Bayesian Cox sensitivity analysis: posterior effects of four admission lipids on recurrence risk (per 1-SD increase)

| **Variable (per 1 SD)** | **Mean β (logHR)** | **95% CrI (β)** | **Median HR** | **95% CrI (HR)** | **Pr(HR<1)** |
| --- | --- | --- | --- | --- | --- |
| Total cholesterol | −0.499 | −1.280 ~ 0.00018 | 0.639 | 0.278 ~ 1.000 | 0.975 |
| HDL-C | −0.489 | −0.724 ~ −0.261 | 0.613 | 0.485 ~ 0.771 | 1.000 |
| Triglycerides | −0.189 | −0.415 ~ 0.024 | 0.830 | 0.661 ~ 1.020 | 0.959 |
| LDL-C | −0.008 | −0.231 ~ 0.198 | 0.995 | 0.794 ~ 1.220 | 0.517 |

Note: Model jointly adjusted for TG, TC, LDL-C, HDL-C and prespecified covariates (gender, smoking, drinking, diabetes, hypertension, gout, hepatic steatosis, first episode, lipid-lowering therapy, RAC severity, age, BMI, and CRP). β, denotes log(HR); CrI, credible interval; Pr(HR<1), posterior probability that HR<1 (β<0).

Supplementary Table 4. Restricted mean recurrence-free survival time (RMST) within 12 months by admission HDL-C levels

| Variables | RD at 6 mo (95% CI) | RD at 12 mo (95% CI) | ΔRMST at 12 mo (95% CI) | P |
| --- | --- | --- | --- | --- |
| HDL-C, tertiles |  |  |  |  |
| T3 | Reference | Reference | Reference |  |
| T1 | 21.3% (13.7–28.8) | 32.6% (22.9–42.4) | –2.22 (–2.85, –1.59) | <0.001 |
| T2 | 4.4% (–1.0–9.7) | 11.5% (2.7–20.3) | –0.49 (–0.92, –0.06) | 0.026 |

Supplementary Table 5. Additive interaction analysis between low HDL-C and metabolic comorbidities on recurrence risk

| Interaction variable | HR_10_ | HR_01_ | HR_11_ | RERI (95% CI) | AP | S |
| --- | --- | --- | --- | --- | --- | --- |
| Diabetes | 2.61 (1.33 ~ 5.13) | 0.98 (0.30 ~ 3.19) | 3.17 (1.57 ~ 6.39) | 0.57 (–0.93, 2.08) | 0.18 | 1.36 |
| Hypertension | 2.82 (1.50 ~ 5.32) | 0.92 (0.25 ~ 3.37) | 2.48 (1.15 ~ 5.35) | –0.26 (–2.06, 1.54) | –0.11 | 0.85 |
| Hepatic steatosis | 2.44 (0.83 ~ 7.17) | 0.83 (0.25 ~ 2.68) | 2.43 (0.88 ~ 6.68) | 0.16 (–1.36, 1.69) | 0.07 | 1.13 |
| Gout | 3.07 (1.63 ~ 5.76) | 2.27 (0.61 ~ 8.42) | 3.83 (1.51 ~ 9.71) | –0.51 (–4.38, 3.36) | –0.13 | 0.85 |
| First episode | 1.50 (0.63 ~ 3.55) | 0.29 (0.10 ~ 0.84) | 1.11 (0.47 ~ 2.61) | 0.33 (–0.45, 1.10) | 0.29 | –0.51 |

HR_10_: low HDL-C without comorbidity; HR_01_: high HDL-C with comorbidity; HR_11_: low HDL-C with comorbidity; RERI: Relative Excess Risk due to Interaction; AP: Attributable Proportion due to interaction; S: Synergy Index.
